# Interferon Gamma Release Assays for the Diagnosis of Latent TB Infection in HIV-Infected Individuals in a Low TB Burden Country

**DOI:** 10.1371/journal.pone.0053330

**Published:** 2013-01-30

**Authors:** Clíona Ní Cheallaigh, Ian Fitzgerald, Jacinta Grace, Gurmit Jagjit Singh, Nahla El-Eraki, Noel Gibbons, Joseph Keane, Thomas R. Rogers, Susan Clarke, Colm Bergin

**Affiliations:** 1 Department of Genito-Urinary Medicine and Infectious Diseases, St James’s Hospital, Dublin, Ireland; 2 Department of Clinical Microbiology, St James’s Hospital, Dublin, Ireland; 3 Department of Clinical Medicine, Institute of Molecular Medicine, Trinity College Dublin and St. James’s Hospital, Dublin, Ireland; 4 Irish Mycobacterial Reference Laboratory, Central Pathology Laboratory, St James’s Hospital, Dublin, Ireland; National Institute for Infectious Diseases (L. Spallanzani), Italy

## Abstract

**Background:**

Interferon gamma release assays (IGRAs) are used to diagnose latent tuberculosis infection. Two IGRAs are commercially available: the Quantiferon TB Gold In Tube (QFT-IT) and the T-SPOT.TB. There is debate as to which test to use in HIV+ individuals. Previous publications from high TB burden countries have raised concerns that the sensitivity of the QFT-IT assay, but not the T-SPOT.TB, may be impaired in HIV+ individuals with low CD4+ T-cell counts. We sought to compare the tests in a low TB burden setting.

**Methodology/Principal Findings:**

T-SPOT.TB, QFT-IT, and tuberculin skin tests (TST) were performed in HIV infected individuals. Results were related to patient characteristics. McNemar’s test, multivariate regression and correlation analysis were carried out using SPSS (SPSS Inc). 256 HIV infected patients were enrolled in the study. The median CD4+ T-cell count was 338 cells/µL (range 1–1328). 37 (14%) patients had a CD4+ T-cell count of <100 cells/µL. 46/256 (18% ) of QFT-IT results and 28/256 (11%) of T-SPOT.TB results were positive. 6 (2%) of QFT-IT and 18 (7%) of T-SPOT.TB results were indeterminate. An additional 9 (4%) of T-SPOT.TB results were unavailable as tests were not performed due to insufficient cells or clotting of the sample. We found a statistically significant association between lower CD4+ T-cell count and negative QFT-IT results (OR 1.055, p = 0.03), and indeterminate/unavailable T-SPOT.TB results (OR 1.079, p = 0.02).

**Conclusions/Significance:**

In low TB prevalence settings, the QFT-IT yields more positive and fewer indeterminate results than T-SPOT.TB. Negative results on the QFT-IT and indeterminate/unavailable results on the T-SPOT.TB were more common in individuals with low CD4+ T-cell counts.

## Introduction

Tuberculosis is the most common opportunistic infection in HIV-infected individuals and is responsible for one-third of HIV-associated deaths [1]. HIV increases the risk of re-activation of latent tuberculosis infection (LTBI) from approximately 0.04 cases per 100 person years [2] to as high as more than 10 per 100 person years [3], [4], [5]. Isoniazid preventative treatment of HIV infected individuals with positive tuberculin skin tests (TST) has been shown to reduce the risk of developing active TB by 60% [6], and observational studies have shown an additive benefit when it is used alongside antiretroviral treatment [7], [8], [9].

Until recently, the TST was the only test available to diagnose LTBI. However, the TST has impaired sensitivity in HIV infected individuals [10], [11], and requires a return visit at 48–72 hours: a step with which as few as 30% of HIV infected patients comply [12]. Interferon gamma release assays (IGRAs) are recently developed blood tests that measure *in vitro* responses to mycobacterial RD1 antigens which are unique to mycobacteria in the *Mycobacterium tuberculosis complex* (*Mycobacterium tuberculosis, Mycobacterium bovis, Mycobacterium africanum*) and *Mycobacterium kansasii* and are not present in *M. bovis* BCG [13]. There are two commercially available assays: the Quantiferon 3G In Tube (QFT-IT) assay (Cellestis, Carnegie, Australia), which utilises an ELISA technique to measure the amount of interferon gamma secreted in response to ESAT-6, CFP-10 and TB 7.7; and the T.SPOT-TB (Oxford Immunotec, Abingdon, UK), which uses an ELISPOT to quantify the number of cells producing interferon gamma in response to ESAT-6 and CFP-10. Advantages of IGRAs over TSTs in HIV infected individuals include an internal control for false negative results and the need for only one visit to test. In addition, it has been reported that the effect of HIV infection on the interferon gamma response to RD1 antigens is less than on the response to the PPD proteins used in the TST: the mechanism underlying this observation is unknown [14].

Which interferon gamma release assay to use in HIV infected individuals remains a matter of debate. In the absence of a gold standard test for LTBI, it is difficult to compare the sensitivity and specificity of the assays. CD4+ T-cells, which are depleted in advanced HIV, are responsible for producing interferon gamma in response to RD1 antigens [15] and there is concern that low CD4+ T-cell levels may impair the sensitivity of IGRAs, particularly the QFT-IT assay [16]. The T-SPOT.TB is thought to be less susceptible to this effect as the number of peripheral mononuclear cells in the assay is standardised. Studies to date of one or both IGRAs for the diagnosis of latent tuberculosis in HIV infected individuals have shown conflicting results regarding the impact of low CD4+ T-cell counts on the tests: a number of studies from high and low TB prevalence regions have found that the sensitivity of the QFT-IT and earlier versions of the Quantiferon, but not T-SPOT.TB is impaired in those with advanced immunosuppression [17], [18], [19], [20]; however, another study from a region of low TB prevalence observed that T-SPOT.TB but not QFT-IT sensitivity was impaired in those with low CD4+ T-cell counts [21]. A number of recent metanalyses found that HIV-associated immunosuppression, measured as CD4^+^ T-cell count, negatively affects the performance of QFT-IT, and to a lesser extent, T-SPOT.TB [22], [23].

We sought to assess the performance of the two commercially available assays, Quantiferon 3G In Tube and T-SPOT.TB, in a HIV outpatient clinic in a low TB prevalence environment in which HIV infected individuals had varying degrees of exposure to active tuberculosis and a wide range of CD4+ T-cell counts. We sought to assess 1) the prevalence of positive and indeterminate test results, 2) the correlation of test results with TB exposure, 3) the effect of low CD4+ T-cell counts and HIV viraemia on test results and 4) inter-test agreement.

## Methods

### Study Setting

The study was carried out at the HIV outpatient clinic in St James’s Hospital, Dublin, Ireland which provides care to approximately 1700 HIV+ individuals/annum (Helen Tuite, personal communication). The incidence of tuberculosis in Ireland is 11/100,000 per annum [24]. On average, 8 cases of active tuberculosis in HIV infected patients are treated in St James’s per annum, giving an incidence rate of 320/100,000 per annum (unpublished data).

### Study Participants

The study was approved by the Joint Research Ethics Committee of St James’s Hospital, Dublin in accordance with the Helsinki Declaration. All patients provided written informed consent. HIV-1 infected patients were recruited from outpatient HIV clinics from August 2008 to February 2010. Patients with current active tuberculosis were excluded. Patients underwent testing with the TST, QFT-IT and T-SPOT.TB assays. Patients also completed an investigator-administered questionnaire on previous history of tuberculosis (active or latent) and risk factors for TB exposure. Countries with annual incidence of TB >100/100,000 as per the WHO database [25] were classified as countries of high TB prevalence. CD4+ T-cell counts and HIV viral loads were obtained from medical records. In post-hoc analysis, patients with a previous diagnosis of tuberculosis were excluded from the main analysis. Their data is presented separately in supplemental information.

### Laboratory Tests

Peripheral blood samples were obtained simultaneously for the QFT-IT and T-SPOT.TB tests. The medical scientist who performed the tests was blind to patient characteristics.

### T-SPOT.TB

The T-SPOT.TB test was performed as previously described and according to manufacturer’s instructions [26]. Spot-forming units were counted by a trained medical scientist using a standardized magnifying lens. The T-SPOT.TB was considered reactive if the response to either ESAT-6 or CFP-10 minus the negative control was ≥6 spot forming cells, or >2 × the negative control. The result was considered indeterminate if the reading in the negative control was >20 spot forming cells or if the reading in the positive control was <20 spot forming cells.

### QFT-IT

For QFT-IT, 1-ml aliquots of blood were collected in each of three tubes, one coated with ESAT-6, TB 7.7 and CFP-10 along with a negative and positive control tube. The test was performed as previously described and per the manufacturer’s instructions [27]. The QFT-IT was considered positive if the interferon-γ response to TB antigens minus the negative control was ≥0.35 IU/ml and >25% of the negative control; negative if these criteria were not met; and indeterminate if either the negative control had a result of >8 IU/ml or the positive control had a result of <0.5 IU/ml. 10 IU/ml is the upper limit of the assay, and values of >10 IU/ml were recorded as 10 IU/ml.

### Tuberculin Skin Test

A TST was part of the initial study protocol. 2 tu of PPD (Statens Serum Institut) were injected intradermally and the diameter of induration was measured by a trained clinical nurse specialist at 48–72 hours. Because of a poor rate of return for measurement of induration and of high rates of refusal to participate in the study because of the requirement for the TST, we changed study protocol in July 2009 and decided to only carry out IGRA testing on the remaining patients.

### Positive Test Results

Patients with positive results on QFT-IT, T-SPOT.TB or TST were informed about their positive test results, and were offered isoniazid preventative treatment.

### Statistical Methods

Data were entered into an Access database (Microsoft, Redmond, VA) and were analysed using SPSS (SPSS version 16.0, SPSS Inc). Outcomes included prevalence of positive test results, concordance between tests, factors associated with positive and indeterminate test results. McNemar’s test was used to compare the prevalence of positive and indeterminate test results between the two IGRAs. Logistic regression was used to calculate odds ratios. Other studies have reported an association between low CD4 T-cell counts and negative IGRA results. In our patient group, CD4+ T-cell counts were associated with other factors associated with TB exposure, e.g. patients from countries of high TB prevalence had a higher median CD4+ T-cell count. Therefore, we used multivariate forwards stepwise binomial logistic regression analysis to control for potential confounding variables. Predictor variables included in the model were age, gender, origin from a country of high TB prevalence, previous TB exposure and injection drug use. As homelessness and incarceration were highly correlated with injection drug use, and residence in a country of high TB prevalence was highly correlated with origin from a country of high TB prevalence; homelessness, incarceration and residence in a country of high TB prevalence for more than two months were not included in the multivariate model. CD4+ T-cell count was analysed as a continuous variable, and was transformed using square root to normalise data. Results are presented as adjusted odds ratios with 95% confidence intervals. Kendall’s rank correlation was used to assess correlation between interferon gamma responses and CD4+ cell counts. Cohen’s kappa was used to assess inter-test agreement. Indeterminate IGRA results were excluded from the logistic regression analysis and analysis of inter-test agreement, but included in the correlation analysis. A *P*-value <0.05 (two-tailed) was taken as the level of significance.

## Results

### Study Population

256 HIV+patients were recruited to the study. Baseline characteristics of the patients are presented in table 1. 85/256 (33%) individuals were from countries of high TB prevalence (incidence >100/100,000). 171 (67%) were from countries of low-moderate TB prevalence*;* of these, 112 (65%) reported one or more risk factors for TB exposure. Only 59 individuals (23% of total) reported no risk factor for exposure to TB.

**Table 1:** Baseline characteristics of study population (n = 256).

Patient Characteristics	Numbers and Percentages
**Age**	
Median and range	36 years (17–66)
**Gender**	
Male	150 (59%)
Female	106 (41%)
**Geographic Origin**	
Irish or other Western European	154 (61%)
Sub-Saharan African	80 (31%)
South-East Asian	5 (2%)
Eastern EuropeanOther	10 (4%)7(2%)
**HIV History**	
CD4+ T-cell count, median and range	338 (1–1328)
CD4+ T-cell count<50×10?6/L	22 (9%)
CD4+ T-cell count <100×10?6/L	37 (15%)
CD4+ T-cell count <250×10?6/L	96 (38%)
Patients on HAART	177 (69%)
Viral load <50 copies/ml	127 (50% of total, 72% of those on HAART)
**Tuberculosis History**	
Prior diagnosis of latent tuberculosis infection	7 (2%)
History of BCG vaccination (of those with known BCG status)	169/194 (*88%)*
BCG vaccination status unknown	62/256 *(23%)*
Currently or previously incarcerated	43(16%)
Current or previous injection drug use	71 (29%)
Current or previous homelessness	30 (12%)
Current or previous occupational exposure to tuberculosis	15 (6%)
2 months or more in region of high TB prevalence (if origin from country of low TB prevalence)	10/160 (6%)
Self-reported close contact with case of active tuberculosis	61 (24%)

CD4+ T-cell counts were measured concomitantly with IGRA tests in 91% of patients. In the remaining 9%, the median number of days between CD4+ T-cell count measurement and IGRA testing was 64, range 6–150. 70% of HIV viral load measurements were concomitant with IGRA testing. In the remaining 30%, the median number of days was 40, range 6–343.

256 patients had blood drawn. The T-SPOT.TB could not be carried out on 9 samples: in 6 cases because there were insufficient mononuclear cells and in 3 cases because the sample had clotted. The QFT-IT assay was done on all 256 samples.

46/256 (18%) of QFT-IT results were positive and 204/256 (80%) were negative. 28/247(11%) of T-SPOT.TB results were positive, and 201/247(81%) were negative. The QFT-IT yielded more positive results (p = 0.01). The QFT-IT yielded more positive results than the T-SPOT.TB irrespective of geographic origin (11% vs 5% in those from countries of low-moderate TB prevalence, 0 = 0.02; 32% vs 22% in those from countries of high TB prevalence, p = 0.04).

### Indeterminate Results

The QFT-IT yielded fewer indeterminate results than the T-SPOT.TB (2% vs 7%, p = 0.04). 6 patients had an indeterminate QFT-IT result, of whom 4/6 (67%) had a mitogen of <0.5 IU/ml, and 2/6 (33%) had a mitogen of >0.5 IU/ml but a nil response that was greater than the mitogen. 18 patients had an indeterminate T-SPOT.TB result, of whom 9/18 (50%) had a positive control of <20 cells, and 9/18 (50%) had a negative control of >10 cells. As noted above, the T-SPOT.TB could not be carried out in 9 additional patients. There were insufficient indeterminate QFT-IT results for regression analysis. On univariate regression analysis of indeterminate T-SPOT.TB results, there was no statistically significant association with CD4+ T-cell count (OR 1.04, p = 0.15); however, if those with insufficient cells to carry out the T-SPOT.TB were pooled with indeterminate results, there was a statistically significant association between CD4+ T-cell count and indeterminate results (OR 1.079, p = 0.02). Individual indeterminate QFT-IT and T-SPOT.TB results are detailed in tables S3 and S4.

### Association between IGRA Results and TB Exposure

On multivariate regression analysis, positive QFT-IT and T-SPOT.TB results were associated with geographical origin from countries of high TB prevalence (OR 4.05, p 0.002 for QFT-IT and OR 4.44, p 0.006 for T-SPOT.TB). Other variables associated with possible exposure to TB such as injection drug use and incarceration, as detailed in Table 2 and 3, were not statistically significant predictors of results.

**Table 2:** Association between positive QFT test results and patient variables.

	Univariate Analysis			Multivariate Analysis		
Predictor variable	Univariate OR	95% CI	P value	Adjusted OR	95% CI	P value
Gender	1.336	.703–2.5	.376	.746	.361–1.541	.428
Age	.975	.938–1.013	.421	.973	.933–1.015	.201
Origin from country of highTB prevalence	3.58	1.85–6.93	0.000	4.053	1.698–9.676	.002
Previous exposure to TB	1.66	.822–3.335	.158	1.650	.772–3.526	.196
>2 months in country of high TBprevalence (in individuals fromcountries of low TB prevalence)	0.00	0.00–0.00	.999			
Occupational exp	0.939	.253–3.475	.924	.675-.	167–2.719	.580
IDU	2.076	.913–4.720	0.081	1.197	.426–3.364	.733
Homelessness	3.557	0.814–15.535	.092			
Prison	2.392	0.806–7.099	0.116			
ART	1.045	.470–2.322	0.915	.912	.386–2.158	.834
CD4+ T-cell count	1.055	1.006–1.107	0.027	1.069	1.009–1.131	0.022
HIV viral load <50 copies/ml	0.663	0.347–1.268	0.215	1.349	.666–2.734	.406

**Table 3:** Association between positive T-SPOT.TB test results and patient variables.

	Univariate			Multivariate		
Predictor variable	OR	95% CI	P value	OR	95% CI	P value
Gender	1.367	.617–3.026	.441	.746	.300–1.854	.528
Age	.982	.937–1.029	.439	.971	.923–1.021	.257
Origin from country of highTB prevalence	4.845	2.075–11.31	<0.001	4.443	1.535–12.857	.006
Previous exposure to TB	1.512	.641–3.566	.345	1.561	.610–3.997	.353
>2 months in country of highTB prevalence (if from countryof low TB prevalence)	1.265	.565–2.832	.568			
Occupational exposure	1.946	0.511–7.412	.329	.791	.174–3.600	.762
IDU	.213	0.049-.930	.040	.485	.090–2.613	.400
Homelessness	.272	.035–2.1	.212			
Prison	.390	.088–1.729	.215			
ART	.733	.263–2.047	.554	.411	.130–1.302	.131
CD4+ T-cell count	1.007	.951–1.067	.803	1.005	.939–1.076	.877
HIV viral load <50 copies/ml	1.624	.725–3.641	.239	1.985	.441–8.928	.371

### Effect of CD4+ T-Cell Count and HIV Viral Load on Positive Test Results

On univariate and multivariate regression analysis, excluding indeterminate results, we found a statistically significant association between low CD4+ T-cell count and negative QFT-IT (OR 1.055, p = 0.03, Table 2) but not T-SPOT.TB results (OR 1.005, p = 0.9, Table 3). To confirm this result, we used Kendall’s correlation analysis to assess the correlation between CD-4 T-cell count and the interferon gamma response to TB antigens and to the positive control in both tests. In the QFT-IT assay, there was a significant correlation between CD4+ T-cell count and interferon-γ response to TB specific antigens (correlation co-efficient 0.201, p<0.001). There was no significant correlation between the CD4+ T-cell count and interferon-γ response to the mitogen positive control (correlation co-efficient 0.047, p = 0.3). It is important to note that the upper limit of the interferon-γ ELISA is 10 IU/ml, which may affect interpretation of this data. In the T-SPOT.TB assay, there was no statistically significant correlation between the CD4+ T-cell count and interferon-γ response to ESAT-6 or CFP 10 (correlation co-efficient 0.057, p 0.236 and correlation co-efficient 0.077, p = 0.104 respectively) or CD4+ T-cell count and response to mitogen (positive control) (correlation co-efficient 0.057, p = 0.266).

An additional 6 patients had insufficient mononuclear cells to carry out the T-SPOT.TB assay. The median CD4 cell count in this group was 200, range 9–613.

### Inter-Test Agreement

Of 43 patients with a positive QFT-IT result, 23 had a positive T-SPOT.TB and 20 had a negative T-SPOT.TB. Of 28 patients with a positive T-SPOT.TB, 23 had a positive QFT-IT and 5 had a negative QFT-IT. 177 patients had a negative result on both tests. Concordance between tests was moderate (kappa = 0.59). On multivariate regression analysis, TB prevalence of country of origin, CD4+ T-cell count and viral load were not associated with discordant results (Table 4). There was a trend towards less discordance in individuals from countries with high TB prevalence.

**Table 4:** Factors associated with discordant IGRA results.

Risk Factor	Unadjusted OR	95% CI	P value	Adjusted OR	95% CI	P value
Origin from country of high TB prevalence	0.67	0.289–1.556	0.352	0.548	.218–1.375	0.200
CD4+ T-cell count	1.042	0.980–1.108	0.188	1.046	.979–1.117	0.186
HIV viral load <50	0.786	0.340–1.814	0.572	0.852	0.356	2.040

Multivariate model also included age and gender as independent predictor variables.

### Tuberculin Skin Test Results

A TST was part of the initial study protocol. Of the first 180 patients, 162 had a TST placed. 69/162 (43%) failed to return to have their reaction measured. 9/93 (10%) TSTs which were measured were positive (see tables S1 and S2).

### Individuals with Previous Active Tuberculosis

An additional 34 individuals who reported having previously been diagnosed with tuberculosis, and who had successfully completed treatment for tuberculosis at least 6 months prior to recruitment were also tested using the QFT-IT and T-SPOT.TB. 19 were male, and 23 were from countries of high TB prevalence. The CD4+ T-cell counts of these individuals ranged from 8–950, with a median CD4+ T-cell count of 307. 5/34 had a CD4+ T-cell count of less than 100.

13/34 (38%) had a positive and 21/34 (62%) had a negative QFT-IT. 8/34 (24%) had a positive and 26/34 (76%) had a negative T-SPOT.TB. On multivariate regression analysis previous active TB was not a statistically significant predictor of a positive result on either QFT-IT or T-SPOT.TB: adjusted OR 1.76, 95% CI 0.78–3.99, p = 0.17 for QFT-IT and adjusted OR 1.56, 95% CI 0.60–4.08, p = 0.36 for T-SPOT.TB.

Interestingly, when these individuals were pooled with the other participants in the study, on multivariate regression analysis of the effect of CD4+ T-cell count on positive results, CD4+ T-cell count was no longer attained statistical significance as a predictor of QFT-IT results (unadjusted OR 1.044, adjusted OR 1.042, 95% CI.996–1.091, p = 0.076).The lack of a statistically significant association between CD4+ T-cell count and T-SPOT.TB results remained unchanged (unadjusted OR 1.007, adjusted OR 1.003, 95% CI.949–1.060, p = 0.931).We hypothesise that individuals with previous active tuberculosis may have higher numbers of circulating TB-antigen specific effector T-cells, and that the impact of low CD4+ T-cell counts on the QFT-IT in this setting is attenuated.

## Discussion

Effective treatment of latent tuberculosis in HIV infected individuals offers an opportunity to prevent significant morbidity and mortality. However, accurate diagnosis of LTBI in HIV infected individuals is challenging. Our experience with the TST highlighted its limitations in our setting: 43% of patients who had a TST failed to return for test reading, and thus had no result available. Interferon gamma release assays require a single visit, and thus offer a clear practical advantage over the TST.

Which interferon gamma release assay to use in HIV infected patients is currently under debate. While longitudinal data will inform us as to sensitivity and specificity of the tests in identifying individuals at risk of developing active TB disease, clinicians and laboratories need interim data on which to base a decision regarding which test to use. Previous studies in countries of high TB prevalence [17], [18], had raised concerns about an increased rate of negative QFT but not T-SPOT.TB results in individuals with low CD4+ T-cell counts; however, a recent metanalysis called this into question [28] Our clinical cohort offered an opportunity to directly compare current commercial versions of the tests in an outpatient clinic in a low TB prevalence setting with individuals with a range of CD4+ T-cell counts.

We found more positive results with the QFT-IT In Tube (QFT-IT) assay than with the T-SPOT.TB: 20% of QFT-IT results were positive compared to 13% of T-SPOT.TB results. Our results contrast with previous comparisons of the two tests which reported a higher rate of positive tests with the T-SPOT.TB than previous versions of Quantiferon [18], [29], but agree with a comparison [17] which also used the most recent version of the assay, Quantiferon Gold In Tube. The Quantiferon Gold In Tube differs from the previous version of the Quantiferon in that it includes an additional TB antigen, TB7.7, and in that blood is drawn directly into tubes coated with TB antigens, ensuring immediate T-cell stimulation. This may explain why this assay gives more positive results and fewer indeterminate results than previous versions of the Quantiferon assay, and why its performance relative to the T-SPOT.TB is improved. In addition, the time lag between drawing of blood and performance of the T-SPOT.TB assays in a clinical setting like ours may impair T-SPOT.TB performance.

When used to diagnose LTBI, rates of indeterminate results with the QFT-IT have ranged from <1–11%, and rates of indeterminate results with T-SPOT.TB have ranged from <1–14% as reviewed by Cattamanchi and colleagues [28]. We found a statistically significantly lower rate of indeterminate results with QFT-IT than T-SPOT.TB: 2% of QFT-IT results were indeterminate, compared to 7% of T-SPOT.TB results.

Individuals with advanced immunosuppression are those most at risk of re-activation of LTBI and are a key priority for diagnosis and treatment. Previous reports from Sub-Saharan Africa [17], [18] found trends towards markedly lower levels of positive Quantiferon/QFT-IT but not T-SPOT.TB results in individuals with CD4+ T-cell counts of <100, whereas other studies from low TB prevalence countries had not seen such a marked trend [21]. A number of papers have reported an increased rate of lack of response to PHA in HIV-infected individuals with low CD4+ T-cell counts [17], [21], [30], [31], [32], [33]. Our results confirmed the association between low CD4+ T-cell counts and negative QFT-IT results. We also found that, in those with low CD4+ T-cell counts, the T-SPOT.TB was more likely to have an indeterminate or unavailable result due insufficient cells to carry out the test. Our findings indicate that, in a low TB prevalence setting, HIV+ individuals with advanced immunosuppression are more likely to have negative QFT-IT results. The internal control for false negative results in the QFT-IT does not appear to be sufficient for those with advanced immunosuppression. The internal control for false negative results in the T-SPOT.TB appears to be more sensitive. However, there is a greater number of indeterminate/unavailable results with the T-SPOT.TB and this is also associated with advanced immunosuppression. Both tests may be problematic in those with low CD4+ T-cell counts, and caution is needed in interpreting results in these individuals.

In agreement with other reports, we found inter-test concordance defined as moderate, (kappa of 0.56) between the QFT-IT and T-SPOT.TB assays. It is worth highlighting that 53% patients with positive QFT-IT results had a negative T-SPOT.TB result, and 18% of patients with a positive T-SPOT.TB result had a negative QFT-IT result. Discordant results were not related to advanced immunosuppression. As there is no gold-standard test for latent tuberculosis, longitudinal follow-up is needed to interpret discordant test results.

Differentiating which HIV+ patients are at risk of developing active tuberculosis and who will benefit from preventative treatment from those who are not at risk and do not require preventative treatment is a pressing clinical need. To date, 4 longitudinal studies have assessed the ability of IGRAs to predict which HIV+ patients will develop active TB: 3 with QFT-IT [33], [34], [35] and one with T-SPOT.TB [36]. All reported that patients with positive IGRA results had a similarly higher risk of developing active tuberculosis, although all studies had limitations [28]. The negative predictive value of negative IGRA results appears promising [33], [35], [36], although further large-scale studies are needed to confirm these finding. Research is on-going to find assays which may out-perform currently available interferon gamma release assays. One approach is to use other biomarkers, such as interferon-inducible protein 10, which has been reported to be less affected by CD4+ T-cell counts [37], [38], [39].

In summary, we found more positive and fewer indeterminate results with the QFT-IT than the T-SPOT.TB. Both currently available IGRA tests are affected by low CD4+ T-cell counts: the QFT-IT may give a negative result whereas the T-SPOT.TB may give an indeterminate or unavailable result. We did not find evidence that either test was clearly superior to the other. These findings highlight the need to interpret negative QFT-IT results in these patients with caution and, as stated in recent CDC guidelines [16], to test for latent tuberculosis at higher CD4 counts, if possible. If patients present with advanced immunosuppression, LTBI testing should be repeated once CD4+ cell counts increase.

## Supporting Information

Table S1 **Tuberculin Skin Test Results.** On multivariate regression analysis, a history of injection drug use was associated with failure to return for TST reading, OR 2.30, 95% CI 1.01–5.22, p = 0.05. Of those who returned for TST reading, on multivariate regression analysis there was no statistically significant association between origin from countries of high TB prevalence or previous TB and a positive TST. There was no statistically significant association between CD4 count or HIV viral load and TST results.
(DOCX)

Table S2 **Concordance between TST and IGRA results.** On multivariate regression analysis, there was no statistically significant association between IGRA/TST discordance and country of origin, previous TB, CD4 count or HIV viral load.
(DOCX)

Table S3 **Indeterminate QFT-IT Results.**
(DOCX)

Table S4 **Indeterminate T-SPOT.TB Results.**
(DOCX)
