# Supplementary material for: Interferon Gamma Release Assays for the Diagnosis of Latent TB Infection in HIV-Infected Individuals in a Low TB Burden Country
Source: PLoS One. 2013 Jan 30;8(1):e53330. doi: 10.1371/journal.pone.0053330 (PMC3559731; doi:10.1371/journal.pone.0053330)
Supplement: Table S1 — Tuberculin Skin Test Results. On multivariate regression analysis, a history of injection drug use was associated with failure to return for TST reading, OR 2.30, 95% CI 1.01–5.22, p = 0.05. Of those who returned for TST reading, on multivariate regression analysis there was no statistically significant association between origin from countries of high TB prevalence or previous TB and a positive TST. There was no statistically significant association between CD4 count or HIV viral load and TST results. (DOCX) [file pone.0053330.s001.docx]

| **TST Outcome** | **Numbers and Percentages** |
| --- | --- |
| Recruited while TST part of study protocol | 180/256 (70%) |
| TST placed | 162/180 (63%) |
| Excluded because of previous blistering TST | 18/180 (10%) |
| Failure to return for TST reading | 69/162 (43%) |
| Positive TST | 9/93 (10%) |
| Negative TST | 84/93 (90%) |
