# Supplementary material for: Interferon Gamma Release Assays for the Diagnosis of Latent TB Infection in HIV-Infected Individuals in a Low TB Burden Country
Source: PLoS One. 2013 Jan 30;8(1):e53330. doi: 10.1371/journal.pone.0053330 (PMC3559731; doi:10.1371/journal.pone.0053330)
Supplement: Table S2 — Concordance between TST and IGRA results. On multivariate regression analysis, there was no statistically significant association between IGRA/TST discordance and country of origin, previous TB, CD4 count or HIV viral load. (DOCX) [file pone.0053330.s002.docx]

|  | | **TST positive** | **TST negative** |
| --- | --- | --- | --- |
| **QFT** | positive | 4 (4%) | 16 (17%) |
|  | negative | 5 (5%) | 64 (69%) |
|  | Indeterminate | 0 | 4 (4%) |
| **T-SPOT** | positive | 4 (4%) | 7 (8%) |
|  | negative | 5 (5%) | 71 (76%) |
|  | Indeterminate | 0 | 1 (1%) |
|  | Not processed | 0 | 6 (1%) |
