# Supplementary material for: Interferon Gamma Release Assays for the Diagnosis of Latent TB Infection in HIV-Infected Individuals in a Low TB Burden Country
Source: PLoS One. 2013 Jan 30;8(1):e53330. doi: 10.1371/journal.pone.0053330 (PMC3559731; doi:10.1371/journal.pone.0053330)
Supplement: Table S3 — Indeterminate QFT-IT Results. (DOCX) [file pone.0053330.s003.docx]

**CD4+ count Nil Ag PHA Ag-Nil PHA-Nil Reason for indeterminate**

(10^6^/ml) (IU/ml)

9 0.05 0.1 0.21 0.05 0.16 Mitogen low

13 2.83 0.04 0.51 -2.79 -2.32 Nil higher than mitogen

261 1.71 1.06 0.05 -0.65 -1.66 Mitogen low

301 0.25 0.03 0.03 -0.22 -0.22 Mitogen low

502 1.79 0.59 1.06 -1.2 -0.73 Nil higher than mitogen

608 0.16 0.12 0.04 -0.04 -0.12 Mitogen low
