# Supplementary material for: Interferon Gamma Release Assays for the Diagnosis of Latent TB Infection in HIV-Infected Individuals in a Low TB Burden Country
Source: PLoS One. 2013 Jan 30;8(1):e53330. doi: 10.1371/journal.pone.0053330 (PMC3559731; doi:10.1371/journal.pone.0053330)
Supplement: Table S4 — Indeterminate T-SPOT.TB Results. (DOCX) [file pone.0053330.s004.docx]

**CD4+ Count Nil AgA AgB PHA Reason for indeterminate**

(10^6^/ml) (+cells/ml)

2 0.0 0.0 4.0 1.0 Mitogen low

6 0.0 0.0 0.0 3.0 Mitogen low

93 0.0 0.0 0.0 1.0 Mitogen low

115 0.0 1.0 0.0 0.0 Mitogen low

133 11.0 30.0 52.0 20.0 Nil high

142 11.0 2.0 2.0 20.0 Nil high

169 18.0 27.0 36.0 20.0 Nil high

173 20.0 20.0 20.0 20.0 Nil high

193 0.0 0.0 0.0 3.0 Mitogen low

288 0.0 0.0 0.0 8.0 Mitogen low

342 0.0 0.0 0.0 3.0 Mitogen low

390 27.0 39.0 31.0 20.0 Nil high

401 0.0 0.0 0.0 3.0 Mitogen low

413 0.0 0.0 0.0 0.0 Mitogen low

425 15.0 3.0 5.0 20.0 Nil high

560 53.0 48.0 52.0 20.0 Nil high

619 12.0 10.0 21.0 20.0 Nil high

927 86.0 64.0 55.0 20.0 Nil high
